# The bradykinin-forming cascade in anaphylaxis and ACE-inhibitor induced angioedema/airway obstruction

**DOI:** 10.3389/falgy.2024.1302605

**Published:** 2024-01-25

**Authors:** Berhane Ghebrehiwet, Kusumam Joseph, Allen P. Kaplan

**Affiliations:** ^1^ Division of Rheumatology, Allergy, and Clinical Immunology, SUNY-Stony Brook, Stony Brook, NY, United States; ^2^ Division of Pulmonary and Critical Care Medicine, The Medical University of South Carolina, Charleston, SC, United States

**Keywords:** bradykinin, anaphylaxis, angioedema, gC1qR, IgE

## Abstract

Anaphylaxis is a potentially life-threatening multi-system allergic reaction to a biological trigger resulting in the release of potent inflammatory mediators from mast cells and basophils and causing symptoms in at least two organ systems that generally include skin, lungs, heart, or gastrointestinal tract in any combination. One exception is profound hypotension as an isolated symptom. There are two types of triggers of anaphylaxis: immunologic and non-Immunologic. Immunologic anaphylaxis is initiated when a foreign antigen directly binds to IgE expressed on mast cells or basophils and induces the release of histamine and other inflammatory substances resulting in vasodilation, vascular leakage, decreased peripheral vascular resistance, and heart muscle depression. If left untreated, death by shock (profound hypotension) or asphyxiation (airway obstruction) can occur. The non-immunologic pathway, on the other hand, can be initiated in many ways. A foreign substance can directly bind to receptors of mast cells and basophils leading to degranulation. There can be immune complex activation of the classical complement cascade with the release of anaphylatoxins C3a and C5a with subsequent recruitment of mast cells and basophils. Finally, hyperosmolar contrast agents can cause blood cell lysis, enzyme release, and complement activation, resulting in anaphylactoid (anaphylactic-like) symptoms. In this report we emphasize the recruitment of the bradykinin-forming cascade in mast cell dependent anaphylactic reactions as a potential mediator of severe hypotension, or airway compromise (asthma, laryngeal edema). We also consider airway obstruction due to inhibition of angiotensin converting enzyme with a diminished rate of endogenous bradykinin metabolism, leading not only to laryngeal edema, but massive tongue swelling with aspiration of secretions.

## Introduction

According to the International Consensus of the World Allergy Organization, anaphylaxis is defined as “a life-threatening, generalized or systemic allergic or hypersensitivity reaction that is rapid in onset, and if not attended to immediately may lead to death (1). The initial discovery of “anaphylaxis” is attributed to both Richet and Portier while experimenting to see the effect of toxins produced by jellyfish and sea anemones on animals (1). In an effort to immunize or induce protection (prophylaxis), they injected a dog with the toxin derived from the Portuguese man o'war (2). However, what they found was that instead of protecting the dog as they had predicted, the toxin induced a severe and fatal reaction, a phenomenon which Richet later referred to as anaphylaxis (“no” protection) (2). Indeed, these were the initial observations that laid the foundation for the identification of the molecular players and pathways that are key to triggering and/or exacerbating anaphylaxis. As will be discussed in detail, anaphylaxis is a rapid life-threatening multi-system biologic reaction in response to chemical or biological trigger that induces the release of potent proinflammatory molecules from mast cells and basophils thereby inducing severe respiratory reactions including shortness of breath, urticaria and angioedema (3, 4), laryngeal edema and profound hypotension.

The complement and kallikrein/kinin systems (KKS) are two enzymatic pathways, which upon activation lead to the generation of vasoactive peptides such as the anaphylatoxins (C3a and C5a) and bradykinin (BK) respectively. Either could theoretically contribute to the symptoms of anaphylaxis depending on the initiating stimulus. The role of the bradykinin-forming cascade in hereditary angioedema, particularly C1 inhibitor deficiency has been elucidated in detail, and many excellent reviews regarding pathogenesis have been published (5–7). However, while generally assumed to be true, bradykinin has not been proven to cause angioedema due to angiotensin converting enzyme inhibitors (ACE-inhibitors), and drug trials using agents to block bradykinin have not given clear results (8). However, the difficulty in doing a study of an acute life-threatening angioedema in the emergency room must also be acknowledged. We address the pathogenesis of this entity as well as the treatment of patients; this can be critical since its most common use is as therapy of hypertension and heart disease.

## The bradykinin-forming cascade in angioedema and anaphylaxis

It is common knowledge that the mast cell is a critical initiator of allergic urticaria and angioedema as well as chronic spontaneous urticaria (9). Acute vasoactive substances released from mast cells and basophils include histamine, leucotrienes, and platelet activating factor, while neuropeptides are released from stimulated afferent nerve endings. These include substance P, neurokinin A, and calcitonin gene-related peptide. Substance P is of particular interest since it is an activator of mast cells. Bradykinin formation is basically a consequence of activation of a plasma proteolytic cascade or cellular release of tissue kallikrein and is not typically considered to be a consequence of mast cell activation. However, that view is incorrect. Mast cells contain a particular form of heparin that a highly sulfated; more so than the heparin that is used as an anticoagulant. It is a negatively charged macromolecule (proteoglycan) and can stimulate activation of factor XII and initiate the bradykinin-forming cascade. This ability of mast cell heparin to activate factor XII was reported many years ago (10) and confirmed later using mast cell heparin purified from mastocytomas or lung tissue (11, 12). The heparin derived from mast cells differs from that typically administered for anticoagulation because it has a higher sulfate concentration per mole. This is key for activation of factor XII, known early on to be activated (actually autoactivation in which the surface renders factor XII to be a substrate) by negatively charged surfaces such as a glass test tube (silicates). However the change density and distribution is critical and mast cell heparin fulfills these requirements (13). A second entity, polyphosphate, a prominent secretory product of platelets is also a potent activator of factor XII (14, 15), but its presence and role in mast cells is less clear (16). But *in vitro* studies with purified mast cell heparan reproduces all the kinin producing effects that are requisite.

Significant activation of the bradykinin-forming cascade is not obvious in allergic rhinitis, sinusitis, asthma, or even urticaria and angioedema most likely because the amount of mast cell heparin released is limited to the organ involved although one study reports the presence of cleaved high molecular weight kininogen (HK) in chronic spontaneous urticaria (17) indicative of plasma kallikrein formation and release of bradykinin. The absence of characteristic bradykinin-dependent symptoms such as laryngeal edema or protracted gastrointestinal attacks, suggests that kininase destruction of bradykinin is sufficiently rapid to prevent clinical symptoms in all those entities.

**Figure 1:**
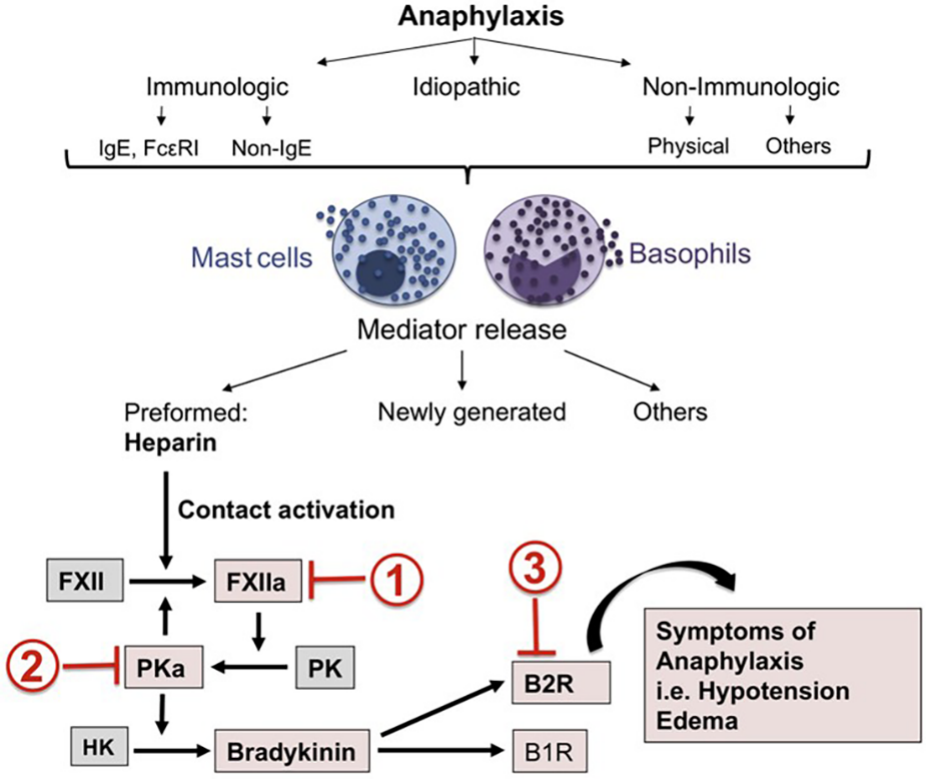
Mast cell activation leads to heparin-dependent initiation of the factor XII-dependent bradykinin-forming cascade leading to symptoms of severe anaphylaxis. Sites of potential inhibition are C1 INH (site 1), Lanadelumab (site 2) or Icatibant (site 3). Reproduced from Sala-Cunill et al. (18).

Anaphylaxis, however, has mast cell activation (as well as basophil activation) potentially throughout the body (18–20) and here is where bradykinin can be an important mediator of symptoms that can be fatal. Laryngeal edema is associated with bradykinin-dependent disorders such as hereditary angioedema (HAE) (primarily due to C1 inhibitor deficiency) or angioedema due to inhibition of angiotensin converting enzyme (ACE), while chronic spontaneous urticaria with or without accompanying angioedema is not associated with laryngeal edema (21, 22). In this respect, anaphylaxis more closely resembles the angioedema of HAE. However, HAE is not associated with hypotension whereas hypotension is characteristic of anaphylaxis and death is due either to shock (anaphylactic shock), asphyxiation due to laryngeal edema, or very severe bronchoconstriction, or a combination of these. In HAE, bradykinin B-2 receptors are present along small venules (23), which is the primary site of action, of bradykinin. Bradykinin is rapidly destroyed upon passage through the pulmonary circulation. In animal models, bradykinin levels on the arterial side of the circulation causes hypotension and blood pressure measurement can actually be used as an assay of arterial bradykinin levels (24). This has not been measured in actual cases of human anaphylaxis but would be of great interest even if it could only be done in a small number of cases. We propose that in anaphylaxis, arterial bradykinin levels may be sufficient to cause hypotension or shock (Figure 1).

Nevertheless although arterial levels of bradykinin have not been measured during anaphylaxis, the entire plasma bradykinin cascade has been shown to be activated (18, 25). The earliest studies suggesting ongoing proteolysis during anaphylaxis have used blood taken from such patients that was anticoagulated, unaware, at the time, that it was due to mast cell heparin (26). Later on, studies using samples of patients who had anaphylaxis in response to insect stings, found that at the peak of an anaphylactic episode in the intensive care unit, the patient's HK was completely cleaved (25) and complexes of Factor XIIa-C1-INH and kallikrein-C1 INH were generated. As has been observed during the earliest studies of anaphylaxis, the plasma partial thromboplastin time (PTT) was markedly prolonged, as if anticoagulated. Decades later, a more complete study of this process demonstrated that human anaphylaxis is associated with activation of Factor XII, conversion of prekallikrein to kallikrein and cleavage of HK to produce bradykinin (18). The initiator was mast cell heparin.

**Figure 2:**
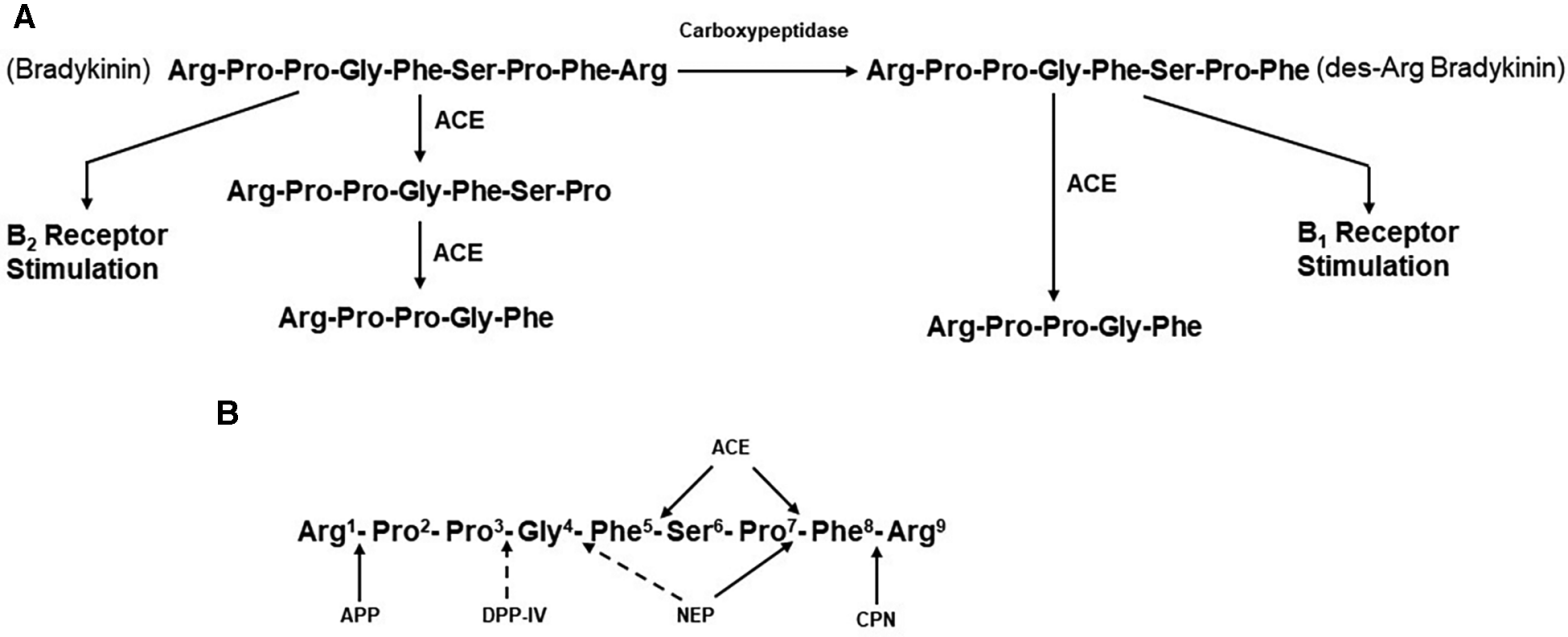
(**A**) metabolism of bradykinin. Bradykinin, a nonapeptide, is digested by angiotensin-converting enzyme (ACE) to release Phe-Arg and then Ser-Pro. These products and the resultant pentapeptide have no interactions with kinin receptors. Carboxypeptidases M, N,(synonymous with carboxypeptidase U) remove C-termainl Arg, leaving an octapeptide (des-Arg^9^-bradykinin) that interacts with bradykinin B1 receptors. ACE degrades des-Arg^9^-bradykinin to a pentapeptide and a tripeptide. (**B**) Enzymatic bond cleavage of bradykinin by tissue and plasma kininases. Carboxypeptidases M and N (**U**) also cleave the C-terminal Arg of bradykinin, B2R, bradykinin B2 receptor; HK, high molecular weight kininogen; CPN, carboxypeptidase N; APP, aminopeptidase P; DPP-IV, dipeptidylpeptidase IV; NEP, neutral endopeptidase; B1R, bradykinin B1 receptor.

The “mediator” of human anaphylactic symptoms is not known and little progress in pathogenesis or therapy has been made during the past 25 years other than the preference for intramuscular epinephrine instead of subcutaneous heparin (27). In spite of the evidence in favor of bradykinin as a major contributor to the symptoms of anaphylaxis, recent review articles barely mention it (3, 28, 29). Platelet activation factor (PAF) is considered to be just such a mediator based on rodent models and human anaphylaxis (30, 31), and deficiency of PAF acetylhydrolase, the enzyme that inactivates PAF, predisposes to severe anaphylaxis. Thus a contribution of PAF to anaphylaxis or even “severe” anaphylaxis cannot be discounted. Yet there are no direct data to implicate PAF as a cause of shock or even hypotension and it is not a known cause of angioedema. Further there is no PAF antagonist available (or on the horizon) to test its contribution, while inhibition of bradykinin and the bradykinin-forming cascade are readily available based on their efficacy for treatment of hereditary angioedema e.g., the B-2 receptor antagonist Icatibant (Figure 2) (32, 33). Administration in this situation can be intravenous (IV); Icatibant was studied IV prior to its approval for subcutaneous administration. Administration of a monoclonal antibody to plasma kallikrein (34, 35) is an alternative. Targeting bradykinin and/or the plasma pathway leading to its formation could be an important addition to the prevention of anaphylactic deaths where symptoms refractory to epinephrine may be encountered (36).

## Bradykinin metabolism: its role in angioedema due to inhibition of angiotensin converting enzyme (ACE)

Angioedema due to ACE-inhibitors accounts for about one-third of acute angioedema cases that present to emergency rooms in the United States (37, 38). ACEIs are used to treat hypertension, for afterload reduction in congestive heart failure, and to prevent acute malignant hypertension (renal crisis) as a manifestation of systemic sclerosis (scleroderma). The overall incidence of ACEI-induced angioedema has been estimated to be between 0.1% and 2% and it is 5 times more common in African Americans than among Caucasians (39). The reaction commonly occurs early in the course of therapy, a few weeks to 4 months after beginning treatment; 50% of cases occur within the first week of treatment (38). However, ACEI-induced angioedema can occasionally occur several years after the start of ACEI therapy. It is important to recognize this possibility because continued use of drugs within this category can lead to increasingly severe attacks and possibly death. Among the manifestations of ACEI-induced angioedema are laryngeal edema and massive tongue or pharyngeal swelling, which can lead to an inability to handle oral secretions, aspiration, or even asphyxiation. Up to 20% of reported cases are life-threatening (40, 41).

Other clinical manifestations of ACEI-induced angioedema include peripheral, orofacial, and gastrointestinal edema (42). Peripheral angioedema can affect the hands, feet, and genitalia (41, 42). Orofacial angioedema can include the face, lips, tongue, pharynx, larynx, and subglottic area. Early laryngeal edema may manifest as hoarseness and progress to inspiratory stridor. Gastrointestinal attacks are due to bowel wall edema and manifest as severe abdominal pain and vomiting, occasionally associated with diarrhea. Attacks of angioedema can last 2 to 4 days.

Although limited in number of subjects, elevated bradykinin levels during attacks of ACE-inhibitor dependent angioedema have actually been documented (43). In addition, the clinical presentation is reminiscent of that seen with hereditary or acquired C1 inhibitor deficiency, in which bradykinin is the primary mediator of swelling (44). The primary substrate of ACE is the decapeptide angiotensin 1, from which it cleaves the C-terminal dipeptide His-Leu to generate the octapeptide angiotensin II (45). ACE is present in plasma. Here, it was originally designated as kininase II (46, 47) because it inactivates the nonapeptide bradykinin by cleaving the C-terminal Phe-Arg, eliminating bradykinin’s ability to interact with either B2 or B1 receptors. A second cleavage removes Ser-Pro, leaving the pentapeptide Arg-Pro-Pro-Gly-Phe (47) (Figure 2A). ACE inhibition, therefore, decreases concentrations of the potent vasoconstrictor angiotensin II, which is beneficial for lowering blood pressure or reducing peripheral resistance to facilitate cardiac output. At the same time, ACE inhibition decreases the degradation of bradykinin, resulting in increased blood levels, of this potent vasodilator.

ACE is known as a dipeptidase, but less appreciated is its function as a tripeptidase, in bradykinin metabolism (47). In a plasma system, the major kininase (i.e, kininase I) is carboxypeptidase N which removes the C-terminal Arg from bradykinin (48). This does not eliminate kinin-like activity, but it does markedly reduce its binding to the B2 endothelial cell receptor, which is constitutively expressed. Instead, the product des-Arg^9^-bradykinin binds to the B1 receptor (49), which is induced in inflammatory states by cytokines such as interleukin-1β (50). In addition, the soluble receptor of the globular heads of the C1q subcomponent of C1 (gC1qR) is released into the pericellular milieu during inflammation or cell activation, has also been shown to induce the B1 receptor (51). Stimulation of B1 receptors by des-Arg^9^-bradykinin leads to vasodilation and increased vascular permeability, similar to that seen with bradykinin stimulation of B2 receptors (52, 53). ACE can then inactivate des-Arg^9^-bradykinin to release Ser-Pro-Phe (i.e., tripeptidase activity) (47) since the Pro-Phe bond can no longer be cleaved when the C-terminal Arg is not present. Thus, it is possible that inhibition of this tripeptidase activity by an ACEI could also lead to accumulation of des-Arg^9^-bradykinin (46), but for this to contribute to angioedema would require upregulation of B1 receptors. The most important site of bradykinin degradation *in vivo* is the pulmonary vasculature (i.e., pulmonary vascular endothelial cells), rather than plasma, and here, ACE appears to be the primary kininase (54, 55). Pulmonary vascular endothelial cells express carboxypeptidase M at the cell surface, which is a different gene product from carboxypeptidase N, but is functionally the same (56).

In the presence of drug-induced ACE inhibition, degradation of bradykinin is dependent on carboxypeptidases such as carboxypeptidases M and N and additional enzymes, including aminopeptidase P (APP), neutral endopeptidase (NEP), and dipeptidyl peptidase IV (DPP-IV) (57–59). The blood and tissue levels of these enzymes may be particularly important in the prevention of angioedema when ACE has been inactivated. The sites of cleavage by each of these enzymes are shown in Figure 2B. Plasma carboxypeptidase N also removes C-terminal Arg from C5a and C3a and is synonymous with the anaphylatoxin inactivator. There is evidence to suggest that African Americans have polymorphisms in the genes that code for APP and/or NEP, resulting in lower blood levels of these enzymes and predisposing those patients taking ACEIs to ACEI-induced angioedema (60). In fact, African Americans have increased cutaneous sensitivity to injected bradykinin even in the absence of medication (60). Furthermore, inhibition of either DDP-IV (59) or NEP (53) in conjunction with ACE inhibition increases the risk of angioedema even further (61).

**Figure 3:**
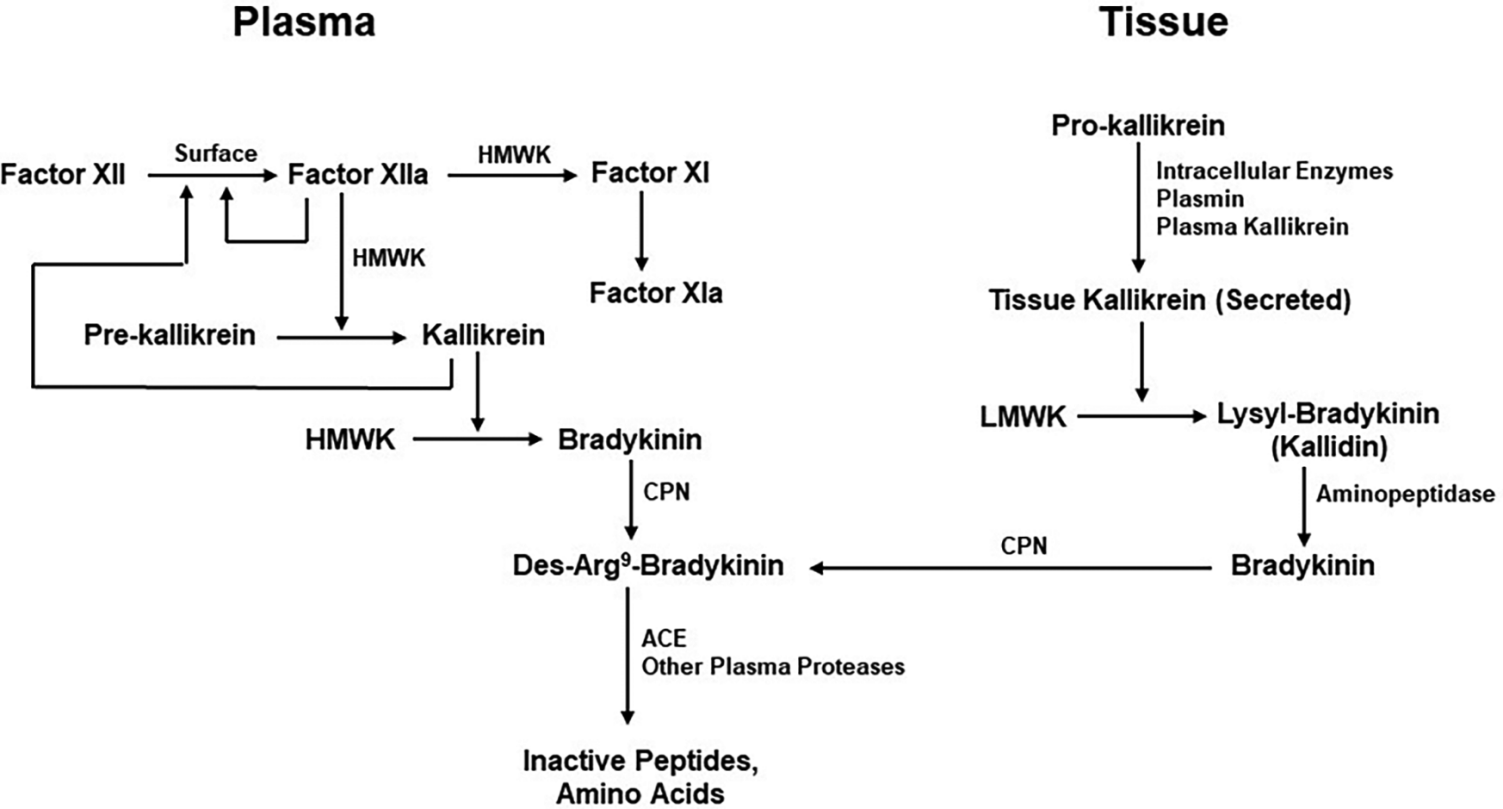
Kinin formation and degradation. Pathways for the production of bradykinin include the plasma (actor XII-dependent intrinsic coagulation pathway) and the extrinsic pathway dependent on secretion of tissue kallikrein. HMWK (or HK), high molecular-weight kininogen; CPN, carboxypeptidase N; ACE, Angiotensin-converting enzyme; LMWK, low molecular weight kininogen.

Bradykinin levels cannot increase unless it is also being continuously produced. Although the mechanisms by which bradykinin is generated are well understood, their individual contributions to maintenance of normal bradykinin levels are unknown. These pathways include the extrinsic pathway dependent on the release of kinin-forming enzymes from cells (tissue) and the intrinsic or factor XII-dependent pathway for bradykinin formation in the blood. The extrinsic pathway involves release of tissue kallikrein (62), a 30kD enzyme that digests a low-molecular-weight form of kininogen (LK) to release lysyl-bradykinin, from which the plasma aminopeptidase P rapidly removes the N-terminal lysine to yield bradykinin (58, 63). The intrinsic pathway involves activation of factor XII, conversion of plasma prekallikrein to kallikrein, and digestion of a high-molecular-weight form of kininogen (HK) by kallikrein to release bradykinin (63) (Figure 3). The baseline bradykinin level in humans is about 10 pg/ml. In a rodent system, deficiency of factor XII decreases baseline bradykinin by one-half (64), indicating that the intrinsic pathway is contributing to the maintenance of that level. The extrinsic pathway may be the other contributor. It should be noted that tissue kallikrein and plasma prekallikrein are encoded by completely different genes and that the difference in amino acid sequence between LK and HK is due to alternative splicing (65) so that they share the same N-terminal half plus bradykinin and the next 9 amino acids. Thereafter, they are synthesized employing different C-terminal domains (exons). One case of total plasma prekallikrein deficiency has had angioedema due to an ACE inhibitor emphasizing the contribution of tissue kallikrein to the level of bradykinin in plasma, or perhaps within the interstitium (66).

## Is there a role for complement or non-IgE-dependent pathways in anaphylactic-like reactions?

The complement system consists of three independent, but cross-reactive pathways and is an important arm of the immune defense mechanism of both humans and animals. It is activated either by foreign antigens, modified self-antigens, or by antigen-antibody complexes, and in the process generates structurally related activation fragments such as C3a, C4a, and C5a. These peptide fragments, or anaphylatoxins (67), share several structural and functional properties with each other. However, C5a and C3a are significantly more active than C4a in their ability to induce the release of histamine from mast cells or basophils (59). Both C3a and C5a can also trigger activation of endothelial cells, and contract smooth muscle cells by binding to their specific receptors. Moreover, anaphylatoxins can activate and induce histamine release from mast cells and basophils and stimulate hydrolytic enzyme release from neutrophils. It is also of particular interest that C3a is selectively chemotactic for eosinophils rather than neutrophils (68) and activates eosinophils to release their constituents at the site of eosinophil accumulation. Together, these can induce vascular permeability, fluid leakage into the interstitium, and contraction of smooth muscle leading to bronchoconstriction. Thus they may have a significant role in non-IgE anaphylaxis in a manner that can be indistinguishable from the classic allergen-driven anaphylaxis (69).

Finkelman FD et al (70). have also reviewed IgE-independent systemic anaphylaxis and clearly describe a role of IgG containing immune complexes in anaphylaxis in guinea pigs and mice that is complement dependent (61). The same may be true in human complement mediated anaphylaxis (71, 72) but a careful reading of the literature indicates that the studies do not always distinguish allergic reactions in which complement has a role from anaphylaxis (69), or assume results in animal models to be true for humans without documentation (71, 72) or are theoretical discussions of what complement activation can do without confirmatory clinical evidence.. Complement activation may lead to elevated blood levels of anaphylatoxins which have been reported to correlate with the severity of human anaphylaxis (3), although measurement of complement components i.e., depletion of C4 or C3 does not reach the predictive value of a tryptase level. Most recently, C5a dependent anaphylaxis in mice was shown to be exacerbated by lipopolysaccharide i.e., preceding bacterial infection (73).

Much progress has been made in recent years regarding the activation of mast cells by non-immune means. Key is the receptor (MRGPRX2), which has many ligands including the nonapeptide substance P, opioids, vasoactive intestinal polypeptide (VIP), and eosinophil granule proteins (69, 74). While agonists such as opioids may augment ongoing allergic reactions, some drugs including fluoroquinolone antibiotics and phenothiazines may serve as initiators. If of sufficient magnitude, such reactions can resemble anaphylaxis, hence the term anaphylactoid reactions. While certain bacterial enzymes can activate the bradykinin-forming cascade (75, 76), and cause hypotension, as is seen in septic shock, anaphylaxis does not appear to be initiated in this fashion.

In the past, intravenously administered contrast agents were a frequent cause of anaphylactoid reactions (77). The osmolarity of these agents appeared to correlate with the incidence of systemic reactions. With newer agents, this has become a rare occurrence. It is of interest, however, that massive enzyme release from blood cells occurred during such anaphylactoid reactions and complement activation was prominent. There need not be activation of the classical complement cascade at the usual initiating step; direct enzymatic activation of C3 and C5 to produce C3a and C5a anaphylatoxins would suffice (78).

It should be mentioned that while we focus on mast cells and or basophils as being the critical cells involved in the pathogenesis (and typically the initiation site) of anaphylaxis, other cells may be contributory. Endothelial cells are intimately involved as the site of vasodilatation and increased vascular permeability at the level of post capillary venules with receptors for histamine and bradykinin, among others (79). Also human vascular endothelial cells appear to be the “sole responder” to anaphylactic mediators as tested *in vitro* (80). Hypotension and shock may occur via a combination of arterial vasodilatation, venular extravasation of fluid, and depressed cardiac function (81). Cells such as neutrophils, eosinophils, or platelets have been noted to be contributory in some instances of non-IgE dependent anaphylaxis however one cannot make any generalization regarding their role if one considers the spectrum of anaphylaxis and focuses on human anaphylaxis only.

### Treatment

When the cause of angioedema is not evident, epinephrine, diphenhydramine, and corticosteroids are usually administered empirically. In the case of ACEI-induced symptoms, a positive response to these agents should not be expected. ACEI-induced angioedema (like all angioedema types mediated by bradykinin) is completely resistant to treatment with antihistamines or corticosteroids. Although often administered, epinephrine is much less reliable in this context than it is in histamine-mediated angioedema. Rapidly accelerating angioedema may be the case and one must be prepared to intubate the patient or perform a tracheostomy even if epinephrine has been administered. There is no specific therapy for peripheral angioedema due to ACEIs, and symptoms of gastrointestinal edema can be treated with intravenous (IV) fluids and analgesics. If ACEI-angioedema is dependent on bradykinin, then approaches that are effective for hereditary angioedema could be employed including infusion of C1 inhibitor (82), which inactivates enzymes involved in bradykinin formation, administration of Ecallantide (83) by subcutaneous injection to inactivate plasma kallikrein, or administration of Icatibant, a reversible bradykinin B2 receptor antagonist (32, 84). Any of these treatments would be reasonable choices for ACEI-induced angioedema, but FDA approval for this indication has not been granted. Since ACEI angioedema is not due to bradykinin overproduction, Icatibant to block bradykinin at the receptor level makes the most sense. In fact, one study of Icatibant therapy for ACE inhibitor angioedema did report excellent results (85). Patients who experience ACEI-induced angioedema may be safely treated with alternative antihypertensive agents such as beta-blockers, calcium channel blockers, or diuretics. ACEIs do, however, have cardioprotective effects shared by angiotensin II receptor blockers (ARBs), which, in many circumstances (e.g., heart failure, diabetes), would be a preferred alternative.

The incidence of angioedema due to ARBs is low (86) (≤10% of that of ACEI-induced angioedema), but it is possible that those who have had an adverse reaction to an ACEI are predisposed to ARB-induced angioedema. Although ARBs do not affect bradykinin metabolism directly, they may raise bradykinin levels (87) by downregulating ACE levels (88), or by sensitizing B2 receptors to the effects of bradykinin (89) through effects on angiotensin II type 2 (AT_2_) receptors (angiotensin’s hypertensive effect is due to interaction with AT_1_ receptors, which are blocked by ARBs). One study suggests that activation of a cellular kininogenase (with kallikrein-like activity) by stimulation of angiotensin II type 1 (AT_1_) receptors may generate bradykinin through cleavage of kininogen (90). Additional mechanisms that are being explored include the possibility that AT_2_-receptor stimulation causes endothelial cell secretion of heat shock protein-90 (HSP-90) (91) or prolyl carboxypeptidase (92) either of which can generate bradykinin by interacting with the plasma prekallikrein-HK complex. Although there are reports of significant angioedema after switching to an ARB from an ACEI (93), most studies indicate that the switch to an ARB is safe and that the benefits outweigh any possible risks (94–96). The renin inhibitor, Aliskaren, does not appear to be associated with any significant incidence of angioedema (97). Idiopathic angioedema should be suspected in any patient who experiences angioedema reactions to both ACEIs and ARBs; such patients may have angioedema independent of any drug exposure. Any positive family history might lead one to consider HAE with normal C1-inhibitor.

## Concluding comments

In contrast to C1 inhibitor deficiency (HAE types I and II), the role of bradykinin is less well defined for ACEI-angioedema, but is generally assumed to be of importance, while its role in causing symptoms of anaphylaxis is not appreciated. For example, bradykinin is not mentioned in a review of progress in understanding anaphylaxis over a 10-year interval; 2013–2023 (98). Thus, the most critical point we wish to make in this manuscript is that there is excellent evidence for activation of the plasma bradykinin-forming cascade in IgE mediated anaphylaxis. The initiator is mast cell heparin (Figure 1), and this will occur with any allergic or non-allergic disorder in which mast cells are activated. That includes allergic rhinitis, extrinsic asthma, chronic spontaneous urticaria, or anaphylaxis. It's a matter of degree and anaphylaxis is most likely to lead to systemic (and local) elevation of bradykinin levels. Because the effects of bradykinin are *not* inhibited by epinephrine, severe anaphylaxis e.g., shock states, will not respond, and this has been observed (99). It may be the key to anaphylactic deaths, which still occasionally occur. Some refer to it as “refractory anaphylaxis” (99). While this is often attributed to inadequate response time to the administration of epinephrine, it need not be so. The assumption that all manifestations of anaphylaxis are responsive to epinephrine administration is likely false even though most manifestations do respond. The many agents available to treat HAE due to C1 inhibitor deficiency may be the answer. For example Icatibant could be administered to block bradykinin and Lanadeumab to prevent further production of bradykinin. Each could be given intravenously (with proper approval for a change in route of administration) however each had originally been considered for intravenous administration until absorption (particularly for Icatibant) was found to be rapid when given subcutaneously. Since angioedema due to ACE inhibitors is a result of inhibition of bradykinin degradation (or perhaps other peptides inactivated by ACE), and not bradykinin overproduction, the only agent used to treat HAE that is likely to be effective is Icatibant.

While entire issues of other journals (JACI, Annals Allergy Asthma, Immunol) have been recently devoted to anaphylaxis as the major emphasis, there appears to be a fear that pointing out what epinephrine cannot do, will lead to a decrease in its use, when an increase in its use is needed. The actual percentage of anaphylaxis cases who receive it may only be 25%–30%. Nevertheless while the morbidity of anaphylaxis is dramatically improved by epinephrine, it is not clear that mortality is affected (99). Our thesis is that mortality is due to vasoactive agents which are not responsive to epinephrine and bradykinin is, perhaps, the leading candidate.
